# Human Bites Over Nose: Management and Reconstruction

**DOI:** 10.1055/s-0042-1760406

**Published:** 2023-03-28

**Authors:** Kuldeep Singh, Krittika Aggarwal

**Affiliations:** 1 Department of Burns and Plastic Surgery, Pt. Bhagwat Dayal Sharma Post Graduate Institute of Medical Sciences (PGIMS), Rohtak, Haryana, India; 2 Department of Burns and Plastic Surgery, Lok Nayak Hospital, Delhi, India

**Keywords:** human bite nose, reconstruction, forehead flap

## Abstract

**Background** Human bite wounds in emergency department need evaluation in regard of reconstruction. These are due to occlusive bite injuries over face. Most commonly, human bites over face involve ear and nose, and may lead to avulsion injury. Defects over nose can be reconstructed immediately after debridement or delayed till the wound heals and scar becomes supple. Thorough wash and lavage with broad- spectrum antibiotic cover has utmost importance in preventing cartilage infection.

**Methods** We report 20 cases of human bite injuries over nose who presented to us in emergency department between 2018 and 2020. At the time of presentation the wound was assessed for closure. If not possible, patient was planned for delayed reconstruction after 3 months. In case delayed reconstruction was planned, the skin and nasal mucosa were approximated at first presentation. The patients underwent paramedian forehead flap after recreation of defect with conchal cartilage graft. Second stage of flap detachment and insetting was done after 3 weeks. After three weeks of second stage, third stage of flap thinning was done. Patients were followed for 3-6 months and subjective satisfaction was noted.

**Results** Nineteen patients underwent delayed staged reconstruction with paramedian forehead flap and one underwent primary wound closure. The flap survival was 100%. The patient satisfaction was excellent in most cases.

**Conclusion** We recommend delayed reconstruction for human bite nasal injuries. For reconstruction, paramedian forehead flap with conchal cartilage graft, if required, provides excellent reconstructive option with good contour and color match and minimal donor site scar.

## Introduction

Bite injuries are a common presentation in the emergency department. It has been documented that human bites constitute 3% of all bite wounds. 1 The most common site is hand, that is, after closed fist injuries. Facial wounds contribute about 15% of all bite injuries. 2 Ear and nose are the most commonly involved structures in human-inflicted facial wounds. Human bite injuries have been divided into closed fist injuries and occlusive bite injuries. 3 In closed fist injuries, the bite wound is a penetrating wound, usually near the metacarpophalangeal joint. The most common complication is tenosynovitis due to high bacterial load in saliva. Occlusive bite injuries occur when there is tissue enclosed between the canine teeth and sufficient force is applied which can cause avulsion injury.

The presentation is usually late. It has been documented that delay beyond 6 to 12 hours of injury increases chances of infection in bite wounds, more so in human bites. 2 However, due to high vascularity of face, the chances are comparatively less in facial bite wounds. 2 Due to the loss of tissue and risk of infection, the management of the wounds is difficult. The timing of reconstruction also needs to be established, keeping in mind the risk of infection. Most of the articles document acute management of human bite injuries over face. This article attempts to outline the acute management and reconstruction of human bite wounds over nose.

## Methods

This is a retrospective review of all the patients who presented to tertiary care hospital in India during 2018 to 2020 with human bite injuries over nose. The institutional ethics committee clearance was obtained (there is no name of the ethics committee in the institution). Written consent was taken from all patients included in the study. The demographic details, associated comorbidities, time since injury, and associated injuries were noted. On local examination of the wound, the presence of bitten off tissue, state of wound margins, structures involved, and presence of debris in the wound were noted. Routine blood investigations and viral markers were done. Anti-rabies and anti-tetanus vaccination were given to all patients. The wound was assessed for possibility of primary closure after debridement and thorough washing. If not possible, the patients were planned for delayed reconstruction after at least 3 months. In case delayed reconstruction was planned, the skin and nasal mucosa were approximated at first presentation. It was done to decrease chances of infection and prevent retraction of mucosa after further healing. The patient satisfaction was noted after 3 months of complete three-stage reconstruction on scale of 1 to 4 (1 - poor, 2 - good, 3 - fair, 4 - excellent). All patients were followed for 3 to 6 months. Table 1 shows the details of all the patients who underwent delayed reconstruction using paramedian forehead flap or primary repair for human bite wounds over nose.

**Table 1:** Details of all the patients who underwent reconstruction/repair for human bite wounds over nose

Serial no	Age	Sex	Time between injury and first stage (mo)	Units requiring reconstruction	Method of repair/reconstruction	Number of stages	Complications	Patient satisfaction	Follow-up time (mo)
1	35	M	3	Tip and left ala	Forehead flap	3	Color mismatch	3	4
2	20	M	3	Tip and left ala	Forehead flap	3	−	4	3
3	40	M	5	Tip and left soft triangle	Forehead flap	3	−	4	3
4	45	F	3	Left ala	Forehead flap	3	−	4	6
5	28	M	At the time of injury	Ala	Primary closure	1	Trapdoor scar	2	3
6	20	F	5	Columella and tip	Forehead flap	3	−	4	5
7	30	M	3	Columella and tip	Forehead flap	3	Mildly acceptable donor site scar	3	5
8	26	M	6	Tip and left ala	Forehead flap	3	−	4	5
9	40	M	3	Right ala, columella and tip	Forehead flap	3	Unacceptable donor site scar	4	6
10	25	M	At the time of injury	Tip	Primary closure	1	Scar hypertrophy	4	3
11	28	F	4	Right ala	Forehead flap	3	−	3	5
12	45	M	3	Tip and columella	Forehead flap	3	−	4	4
13	30	M	3	Right ala	Forehead flap	3	−	4	5
14	25	M	3	Right ala, columella and tip	Forehead flap	3	−	3	5
15	27	M	5	Tip and left ala	Forehead flap	3	−	4	4
16	22	M	3	Left ala	Forehead flap	3	−	4	4
17	20	M	5	Right ala, columella and tip	Forehead flap	3	−	4	5
18	28	M	3	Right ala	Forehead flap	3	−	4	6
19	45	F	4	Tip and left ala	Forehead flap	3	Minimal color mismatch	4	6
20	35	M	3	Tip and left ala	Forehead flap	3	−	4	6

Abbreviations: F, female; M, male.

### Surgical Technique

In case for delayed reconstruction, paramedian forehead flap was planned in first stage with conchal cartilage graft. Fibrotic tissue was excised. In case there was partial loss of any subunit, uninvolved part of the subunit was retained. Template of the defect was made using lint piece. All cases were done after planning in reverse, after recreating the defect. Paramedian forehead flap was raised by the standard technique after marking the defect over forehead. The distal third of the flap was raised subcutaneously, middle third below the frontal muscle, and distal third subperiosteally. The support was provided using conchal cartilage in all cases. Donor site was closed primarily in all cases. The undersurface of the forehead flap was dressed with antibiotic impregnated paraffin gauze which was changed after every 2 days. This raw area healed in the next 10 to 15 days. After 3 weeks, second stage of detachment and insetting was done. Care was taken to achieve eyebrow symmetry. Postoperatively, local hygiene was maintained and local neomycin ointment was applied. Sutures were removed after 7 days. Afterwards, massage was advised. Third stage, that is, flap thinning was done after 3 weeks of second surgery.

## Results

**Fig. 1:**
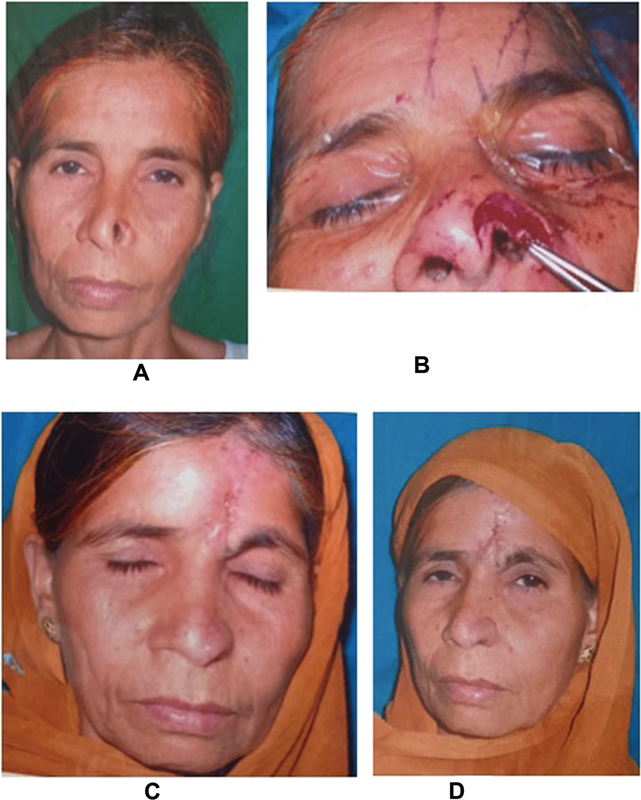
Picture showing actual defect in ( **A** ) and after debridement in ( **B** ). ( **C** and **D** ) Paramedian forehead flap after 7 days of flap detachment and insetting.

The data of patients who presented with human bite injuries over nose between 2018 and 2020 was recorded. The details are as shown in Table 1 . The age of the patients ranged between 20 and 45 years. Most of the patients were male (male-to-female ratio of 16:4). In all the cases the defect was over the distal third of the nose and sometimes included the tip. In one case, suturing of the avulsed tissue was done. Rest of the patients underwent delayed reconstruction. In the first stage, scar excision and recreation of the defect over nose was done and simultaneous paramedian forehead flap cover was done. As cartilage support was needed, conchal cartilage graft was harvested and placed over the defect, under the forehead flap. Donor site over forehead was closed primarily in all cases. The time taken in the first stage ranged from 1.5 to 2 hours. After 3 weeks flap detachment and insetting was done. After another 3 weeks, flap thinning was done in all cases. Flap survival was 100% in all cases. None of the patients had surgical site infection or any such complications. Patient satisfaction was noted after 3 months of final stage and was excellent in most cases (excellent in 15, fair in 4). In one case the satisfaction was good; the patient underwent primary wound repair of the avulsed tissue and developed trapdoor scar over nose. The patient is planned for scar revision with Z-plasty. The donor site scar was acceptable as well. Figs. 1 2 3 4 show preoperative and final photographs for three such cases.

**Fig. 2:**
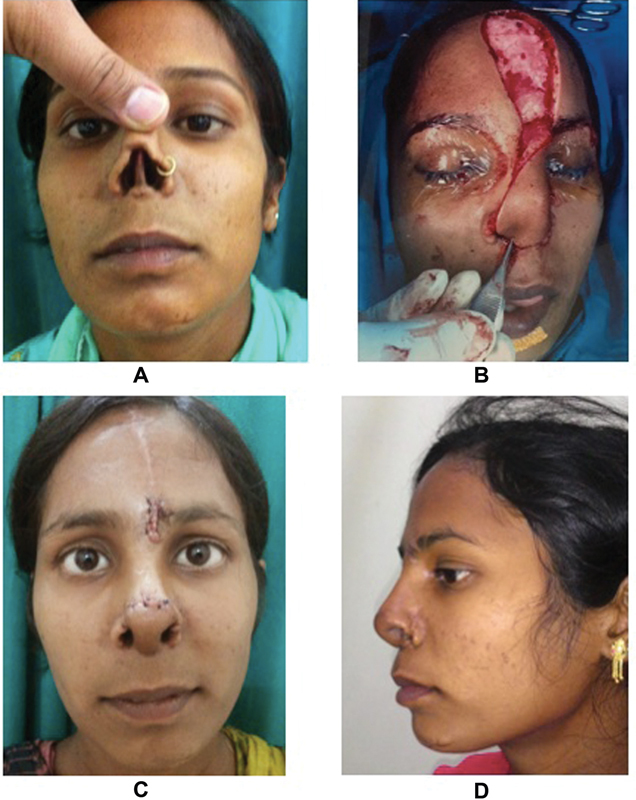
Picture showing actual defect in ( **A** ) and after debridement in ( **B** ). ( **C** and **D** ) Paramedian forehead flap after 7 days of flap detachment and insetting.

**Fig. 3:**
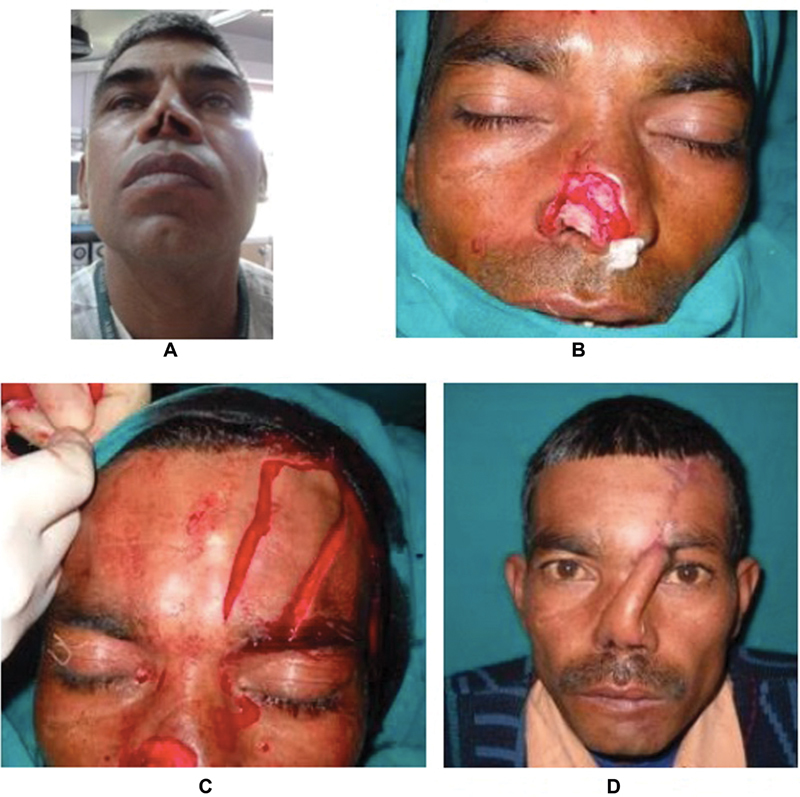
Picture showing preop defect in ( **A** ) and actual defect after debridement in ( **B** ).( **C** ) shows paramedian forehead flap raised after planning in reverse. ( **D** ) shows 100% flap survival with good donor site scar at day 7.

**Fig. 4:**
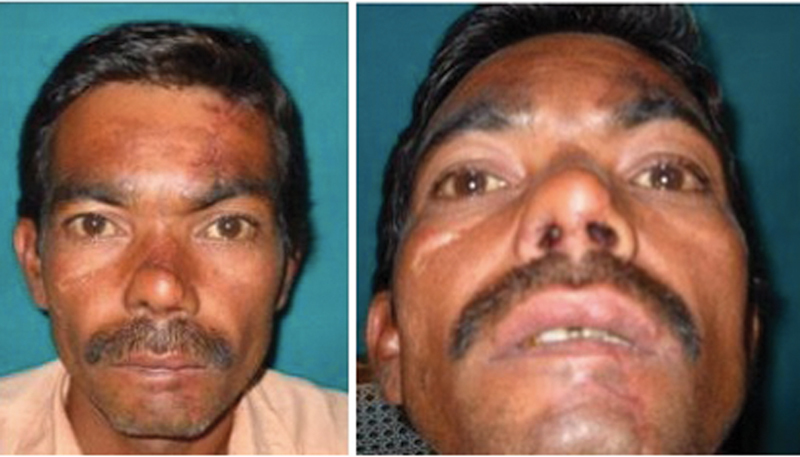
Pictures showing final result after day 5 of flap detachment and insetting. Donor site scar is minimal with excellent contour and color match over nose.

## Discussion

Human bite wounds over face are seen after assault and occur commonly over ear and nose. 2 4 In nose, most common site involved is the distal third. 4 This leads to deformity of tip with or without columella, leading to loss of cartilaginous support and nasal height with visible depression of nose. Most of the times, nasolabial/melolabial flap or forehead flap are used for reconstruction of nasal defects especially those including the tip or ala. 5 Both of these flaps offer good color match and acceptable donor site scars. However, nasolabial/melolabial flap cannot be used for large defects. In such cases paramedian forehead flap is used. Forehead flap can be used for reconstruction of columella, ala, and tip at the same time. The major disadvantage of forehead flap is that it is a staged procedure. After the first stage, the patient needs to tolerate the cosmetic deformity caused, which may lead to social embarrassment. Various modifications of forehead flap have been documented. A Study by Fudem et al 6 documented their results in single-stage forehead flap reconstruction for nose. Fudem et al 6 removed radix, proximal nasal skin, and fat and deepithelialized the proximal pedicle to allow inset without excess compression or kinking . Hsiao et al 7 reported their results of forehead flap for nasal reconstruction in Asians. They noted that Asians have higher chances of scar contracture and pin-cushioning effect. The cartilage framework was noted to be weak in quality and quantity. The modifications reported include extending subunit and flap boundaries, minimizing flap thinning, and overbuilding the nasal framework to combat contraction and suboptimal scarring. 7 Various authors have used pre-expanded forehead flaps as well. 8 9 10 It is well known that the raw area over forehead heals well by secondary intention and leaves imperceptible scar. Hence, donor site does not pose a problem.

It is well documented that human bite injuries have high chances of infection attributing to high bacterial load in saliva. 2 11 The chances of infection are less over face due to high vascularity. Viridans streptococci and staphylococci are commonly in wound isolates. 2 11 *Eikenella corrodens* , a normal inhabitant of the human oral cavity, has a unique association with human bites. 2 11 12 Spread of viral infections like herpes, hepatitis B and C, and human immunodeficiency virus (HIV) is also documented from human bites. 2 3 12 The chances of HIV spread are relatively less. However, these infections should be ruled out in both the individuals—the victim and the one biting him. 2 12 Anti-tetanus vaccination has been advised. Wound debridement with thorough wash is advised. 2 12 Systemic broad-spectrum antibiotics have been advised, especially for fist bite injuries, to decrease infection. Suturing in the presence of overt infection, gross edema, foreign bodies, or visible contamination is not recommended. 2 12 In such cases delayed repair or reconstruction is advised.

The loss of tissue needs reconstruction which can be done immediately after adequate debridement or later. Studies report acute reconstruction using nasolabial or melolabial flap and paramedian forehead flap for nasal defects following human bite injuries. 13 14 15 16 17 However, only few number of cases were reported in such studies. We performed delayed reconstruction of nasal defects to allow the infection to heal and scars to become supple. Though it increases the time the patient has to tolerate the cosmetic deformity, it decreases the chances of infection especially when cartilage graft is to be used. There is abundance of studies reporting the success of forehead flap for nasal reconstruction. However, most of these studies are performed for postmalignancy excision defects and in Caucasian population. This article documents the success and acceptable color match seen after forehead flap cover for post-human bite nasal defects in Indian population.

Human bite wounds are seen in the emergency department and need evaluation for the need of reconstruction. They are due to occlusive bite injuries. Defects over nose can be reconstructed immediately after debridement or delayed till the wound heals and scar becomes supple. We recommend delayed reconstruction to lessen the chances of infection and cartilage absorption as cartilage graft is needed in most cases undergoing reconstruction. Paramedian forehead flap provides excellent reconstructive option with good contour and color match with minimal donor site scar.
